# Pre‐symptomatic transmission of novel coronavirus in community settings

**DOI:** 10.1111/irv.12773

**Published:** 2020-06-19

**Authors:** Dechuan Kong, Yang Zheng, Huanyu Wu, Hao Pan, Abram L. Wagner, Yaxu Zheng, Xiaohuan Gong, Yiyi Zhu, Bihong Jin, Wenjia Xiao, Shenghua Mao, Sheng Lin, Ruobing Han, Xiao Yu, Peng Cui, Chenyan Jiang, Qiwen Fang, Yihan Lu, Chen Fu

**Affiliations:** ^1^ Department of Acute Communicable Diseases Control and Prevention Shanghai Municipal Center for Disease Control and Prevention Shanghai China; ^2^ Department of Non‐communicable Diseases Surveillance Shanghai Municipal Center for Disease Control and Prevention Shanghai China; ^3^ Institute of Communicable Diseases Control and Prevention Shanghai Municipal Center for Disease Control and Prevention Shanghai China; ^4^ Department of Epidemiology School of Public Health University of Michigan Ann Arbor MI USA; ^5^ Department of Epidemiology School of Public Health Fudan University Shanghai China; ^6^ Key Laboratory of Public Health Safety (Ministry of Education) School of Public Health Fudan University Shanghai China; ^7^ Shanghai Municipal Center for Disease Control and Prevention Shanghai China

**Keywords:** China, comorbidity, contact tracing, COVID‐19

## Abstract

We used contact tracing to document how COVID‐19 was transmitted across 5 generations involving 10 cases, starting with an individual who became ill on January 27. We calculated the incubation period of the cases as the interval between infection and development of symptoms. The median incubation period was 6.0 days (interquartile range, 3.5‐9.5 days). The last two generations were infected in public places, 3 and 4 days prior to the onset of illness in their infectors. Both had certain underlying conditions and comorbidity. Further identification of how individuals transmit prior to being symptomatic will have important consequences.

## 1 INTRODUCTION

Novel coronavirus disease 2019 (COVID‐19) was declared a pandemic on March 11, 2020.^1^ Transmission chains have been difficult to identify in routine investigations. Notable cases in Italy^2^ and the United States^3^ had no known source of infection. These cases have raised the concern that there is transmission of the novel coronavirus (SARS‐CoV‐2) in asymptomatic individuals (who never express symptoms) or in pre‐symptomatic individuals (prior to any symptoms).

Many public actions so far, including quarantine measures, body temperature measurement, and fever symptom surveillance, have prioritized identification of possible infected cases. However, these all depend on active expression of symptoms and are not able to identify asymptomatic transmission or pre‐symptomatic transmission.^4^

Better understanding of transmission parameters, including the serial interval and incubation period, can also help determine the possible progression of the outbreak. The serial interval is defined as the interval between a primary case of COVID‐19 developing symptoms and a secondary case developing symptoms, whereas the incubation period is the time lag between infection and the start of symptoms. The Chinese Center for Disease Control and Prevention (CDC) has estimated that the mean serial interval for COVID‐19 is 7.5 days, which is slightly longer than the estimated incubation period of 5.2 days.^5^ However, the serial interval has varied in other studies, such as a median of 4.6 days,^6^ which is shorter than the incubation period.

The challenge for controlling COVID‐19 is to determine at what point an individual becomes infectious, which can have implications for contact tracing and other epidemiological investigations. In addition, one study in China showed that 12.1% of transmission was likely to be pre‐symptomatic.^7^ On February 24, 2020, the China CDC revised the definition of the start date for close contact from “at the illness onset of a confirmed case” to “2 days before the illness onset,”^8^ as increased evidence suggests pre‐symptomatic transmission might be plausible.

Many previous studies of the transmission dynamics of COVID‐19 have relied on publicly available data or large surveillance datasets. It is difficult to discover asymptomatic or pre‐symptomatic cases in these datasets, and overall there is limited information on how to determine when an individual becomes infectious. This study describes a transmission chain of 5 generations involving 10 COVID‐19 cases, and tracks whether infection occurred from asymptomatic or pre‐symptomatic individuals.

## 2 METHODS

An index case (ie, the last case) was first identified in our study who had no obvious previous contact with a symptomatic case of COVID‐19. For the index case, we identified 3 close contacts in the 14 days prior to disease onset, none of which had COVID‐19 at the time of contact. We worked backward to identify several generations of transmission. We used contact tracing to identify possible people who could have exposed the case. Close contacts were defined as people who live, study, work, or otherwise have close contact with the case; medical personnel, family members, or other people who have similarly had close contact with the case and who did not take effective protective measures; other patients and their accompanying staff in the same ward of the case; persons in the same vehicle as the case and who had close contact with the case; and other persons who were deemed close contacts by the field investigator.

We retrospectively summarized the journey and visited places of the cases within 14 days before the illness onset in each generation. In this way, we identified possible source cases and we worked backward to identify cases, which were epidemiologically linked with each other, and which constituted a complete transmission chain. Information about symptoms and date of illness onset was provided directly by the cases. We acknowledge recall bias: Cases may not have been able to remember every contact they had.

All of the investigations were conducted by experienced investigators at Centers for Disease Control and Prevention in the Shanghai Municipality and Zhejiang Province. This study involved the use of existing, routinely collected data from a public health outbreak investigation, under the National Health Commission of the People's Republic of China. Thus, this study is exempt from ethical review and informed consent was not obtained.

## 3 RESULTS

### 3.1 Initiation of investigation

**FIGURE 1:**
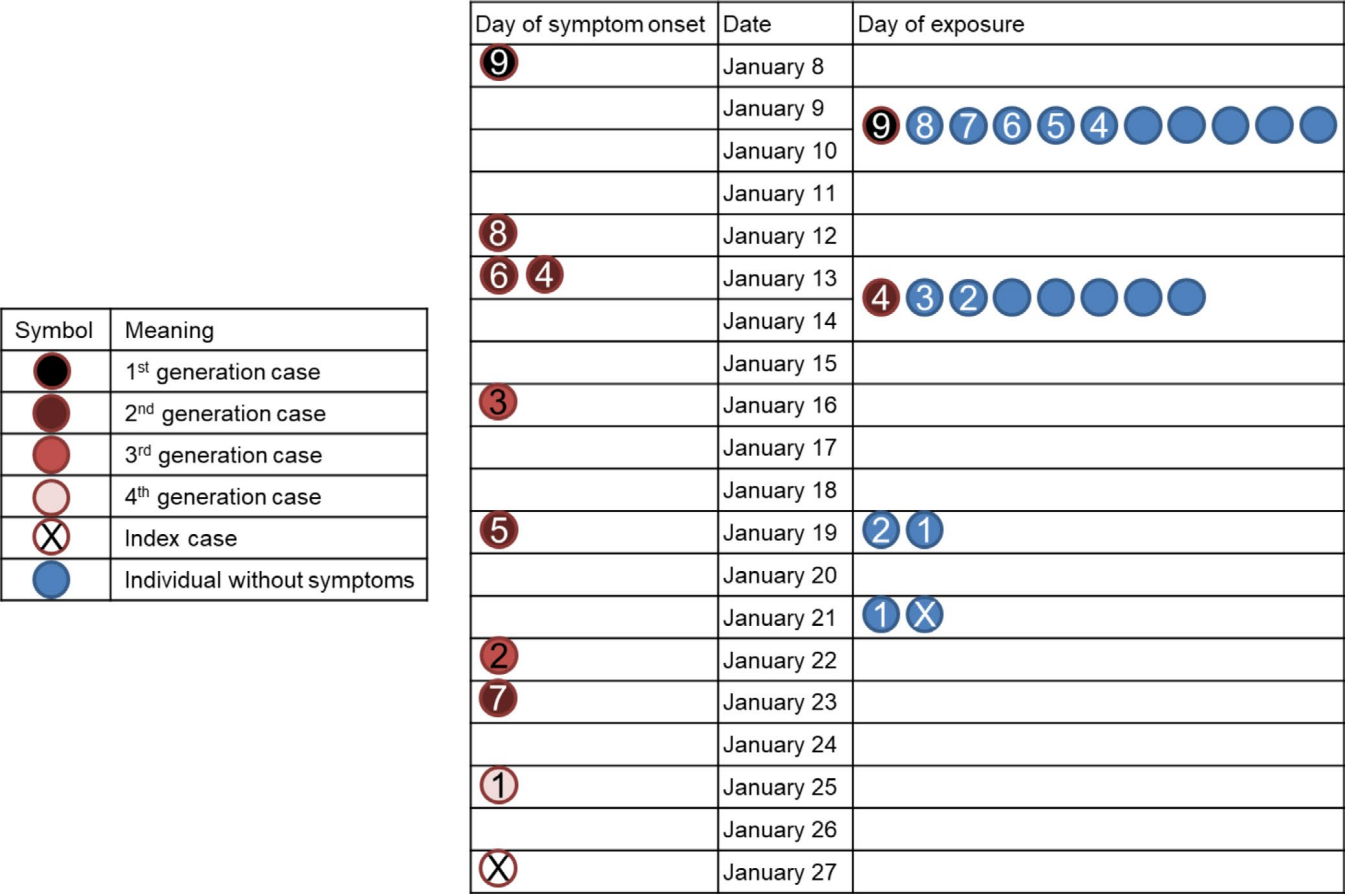
Time line of contact and illness onset of novel coronavirus disease 2019 (COVID‐19) cases in late January 2020. The persons involved in transmission venues are listed, including four dinners (on January 9 and 10, January 13 and 14) and two meetings in public places (on January 19 and 21). Not shown are additional close contacts (with individuals who remained symptom‐free after a 14‐day quarantine): 3 for the index case, 1 for case 1, 1 for case 2, 11 for case 3, 12 for case 5, 1 for case 8, and 1 for case 9

The index case was a 74‐year‐old female who developed symptoms on January 27. She lived alone and had very limited contact with foreign people. She was found to have met with a 75‐year‐old male, in a real estate trade center for 3 hours on January 21, 6 days prior to the onset of her symptoms. The male was recognized as an existing confirmed case (case 1) of COVID‐19, who developed symptoms only on January 25. Subsequently, case 2 was identified, who was a friend of case 1 and had met him for 2.5 hours at a gym on January 19. Case 2 developed symptoms on January 22. The timeline of possible infection and illness onset is illustrated in Figure 1.

### 3.2 Identification of two occasions of pre‐symptomatic transmission

We determined the epidemiological linkages between index case, case 1, and case 2, after excluding all of their close contacts who remained healthy throughout a 14‐day medical observation. It is noted that the infectees (index case and case 1) were possibly infected with SARS‐CoV‐2 by infectors (case 1 and case 2) during the incubation periods of the infectors (Figure 1).

To confirm the finding, we further checked whether there was previous contact between these cases. Cases 1 and 2 had not met for about one month, and did not meet after January 19. Thus, the transmission was likely to occur on January 19 in the gym. The index case and case 1 had also met on January 18. However, case 1 was not infected on that day and thus was likely to have occurred on January 21. Through an examination of cases 1 and 2's clinical symptoms, we inferred both transmitted infection while pre‐symptomatic.

### 3.3 Source of transmission chain

We worked backward to further investigate the chain of transmission. Case 2 and case 3 had not met before; however, they possibly became infected by a common friend (case 4) at dinners on January 13 and 14. Similarly, cases 4‐7 became infected at dinners with a common friend (case 8). During the dinners, case 8's daughter (case 9) could have been the common source of infection, and she was infected by her co‐worker who had traveled back from the city of Wuhan, Hubei Province. Across these cases, we did not recognize any evidence of pre‐symptomatic transmission.

### 3.4 Incubation period and serial interval

In this study, all the cases had an incubation period less than 14 days, with a median of 6.0 days (interquartile range, 3.5‐9.5 days). Comparatively, we calculated the median serial interval to be 5.0 days (interquartile range, 3.0‐5.0 days). For the two instances of pre‐symptomatic transmission, the infectees' contact with the infectors occurred 3 or 4 days prior to the infectors' illness onset, which suggests a shorter latent period than an incubation period.

### 3.5 Co‐morbidities

To identify differences between pre‐symptomatic transmission and symptomatic transmission, we examined the health conditions of all cases. The index case underwent a surgery for stomach cancer and had been receiving chemotherapy for 1.5 years. Case 1 suffered from pneumonia and recovered late December 2019. No other cases had underlying conditions and comorbidity.

## 4 DISCUSSION

In this study, we traced the spread of coronavirus across five generations in Shanghai, China, back to a case who had previously traveled to Wuhan, the original source of the outbreak. The serial interval was estimated to be a little shorter than the incubation period, and there were no known symptoms among suggested possible pre‐symptomatic transmission. However, two infector‐infectee pairs with pre‐symptomatic transmission were identified out of 9 pairs. The index case and case 1 may have been more susceptible to pre‐symptomatic infection due to their health conditions, compared to other cases.

That the latent period may be shorter than the incubation period could affect recommendations for contact tracing and case definitions. We found evidence of pre‐symptomatic transmission 3‐4 days prior to the infectors' illness onset. Thus, we suggest advancing the upper time limit of close contact to 4 days prior to illness onset of a COVID‐19 case, which is greater than the China CDC and WHO guidelines of 2 days.^8^, ^9^ We note that guidelines in Beijing use a time limit of 4 days as of May 18, 2020.^10^ One another study in China reported only 2‐day lead time between infectee's contact with the infector and infector's illness onset,^11^ and we have not found any information that this could be longer. We recognize that increasing the duration of a possible time of infection can add to the workload of routine epidemiological investigations during the epidemic of COVID‐19. Consequently, we recommend it should be applied to cases with an unknown source of infection, such as the index case in our study. Contact tracing guidelines should also rely on the capacity of contact tracing and on updated information on when pre‐symptomatic transmission can occur. For example, a study of COVID‐19 cases in Taiwan also started investigations of COVID‐19 up to 4 days before symptom onset, although this was not consistently done.^12^ Evidence from more chains of transmission can better delineate the borderline between the latent period and infectious period.

Asymptomatic transmission has been documented among returned Japanese citizens from China.^13^ However, it might be misunderstood due to short duration of observation, that is to say, if we extend the duration of observation, we might observe the occurrence of symptoms. Consequently, pre‐symptomatic transmission may be misunderstood as asymptomatic transmission. So far, asymptomatic transmission and pre‐symptomatic transmission have not been well documented and further study of natural history of SARS‐CoV‐2 infection is urgently warranted.

## CONFLICTS OF INTEREST

All authors: No reported conflicts of interest.

## AUTHOR CONTRIBUTION

**Dechuan Kong:** Conceptualization (equal); Investigation (equal); Visualization (supporting); Writing‐original draft (supporting). **Yang Zheng:** Conceptualization (equal); Investigation (equal); Writing‐review & editing (supporting). **Huanyu Wu:** Conceptualization (equal); Funding acquisition (equal); Investigation (equal); Writing‐review & editing (supporting). **Hao Pan:** Conceptualization (equal); Funding acquisition (equal); Investigation (equal); Writing‐original draft (supporting). **Abram L. Wagner:** Writing‐review & editing (supporting). **Yaxu Zheng:** Investigation (supporting); Writing‐review & editing (supporting). **Xiaohuan Gong:** Investigation (supporting); Writing‐review & editing (supporting). **Yiyi Zhu:** Investigation (supporting); Writing‐review & editing (supporting). **Bihong Jin:** Investigation (supporting); Writing‐review & editing (supporting). **Wenjia Xiao:** Investigation (supporting); Writing‐review & editing (supporting). **Shenghua Mao:** Investigation (supporting); Writing‐review & editing (supporting). **Sheng Lin:** Investigation (supporting); Writing‐review & editing (supporting). **Ruobing Han:** Investigation (supporting); Writing‐review & editing (supporting). **Xiao Yu:** Investigation (supporting); Writing‐review & editing (supporting). **Peng Cui:** Investigation (supporting); Writing‐review & editing (supporting). **Chenyan Jiang:** Investigation (supporting); Writing‐review & editing (supporting). **Qiwen Fang:** Investigation (supporting); Writing‐review & editing (supporting). **Yihan Lu:** Investigation (supporting); Visualization (lead); Writing‐original draft (lead). **Chen Fu:** Conceptualization (equal); Funding acquisition (equal); Investigation (equal); Writing‐review & editing (supporting).
